# Development and Evaluation of a Peptide Heterodimeric Tracer Targeting CXCR4 and Integrin α_v_β_3_ for Pancreatic Cancer Imaging

**DOI:** 10.3390/pharmaceutics14091791

**Published:** 2022-08-26

**Authors:** Yaqun Jiang, Yu Long, Hao Ji, Pengxin Qiao, Qingyao Liu, Xiaotian Xia, Chunxia Qin, Yongxue Zhang, Xiaoli Lan, Yongkang Gai

**Affiliations:** 1 Department of Nuclear Medicine, Union Hospital, Tongji Medical College, Huazhong University of Science and Technology, Wuhan 430022, China; 2 Hubei Province Key Laboratory of Molecular Imaging, Wuhan 430022, China; 3 Department of Nuclear Medicine, Zhongnan Hospital of Wuhan University, Wuhan University, Wuhan 430071, China; 4 Key Laboratory of Biological Targeted Therapy, The Ministry of Education, Wuhan 430022, China

**Keywords:** peptide heterodimer, C-X-C chemokine receptor type 4, integrin α_v_β_3_, pancreatic cancer

## Abstract

Nowadays, pancreatic cancer is still a formidable disease to diagnose. The CXC chemokine receptor 4 (CXCR4) and integrin α_v_β_3_ play important roles in tumor development, progression, invasion, and metastasis, which are overexpressed in many types of human cancers. In this study, we developed a heterodimeric tracer ^68^Ga-yG5-RGD targeting both CXCR4 and integrin α_v_β_3_, and evaluated its feasibility and utility in PET imaging of pancreatic cancer. The ^68^Ga-yG5-RGD could accumulate in CXCR4/integrin α_v_β_3_ positive BxPC3 tumors in a high concentration and was much higher than that of ^68^Ga-yG5 (*p* < 0.001) and ^68^Ga-RGD (*p* < 0.001). No increased uptake of ^68^Ga-yG5-RGD was found in MX-1 tumors (CXCR4/integrin α_v_β_3_, negative). In addition, the uptake of ^68^Ga-yG5-RGD in BxPC3 was significantly blocked by excess amounts of AMD3100 (an FDA-approved CXCR4 antagonist) and/or unlabeled RGD (*p* < 0.001), confirming its dual-receptor targeting properties. The ex vivo biodistribution and immunohistochemical results were consistent with the in vivo imaging results. The dual-receptor targeting strategy achieved improved tumor-targeting efficiency and prolonged tumor retention in BxPC3 tumors, suggesting ^68^Ga-yG5-RGD is a promising tracer for the noninvasive detection of tumors that express either CXCR4 or integrin α_v_β_3_ or both, and therefore may have good prospects for clinical translation.

## 1. Introduction

Pancreatic cancer (PC) is a highly malignant phenotype characterized by aggressive invasion and metastasis. Patients with PC often experience few symptoms in the early stages, and in most patients, local invasion and distant metastases are typically observed at diagnosis and therefore are unresectable [1,2,3]. Currently, radical surgery at the early stages is the only opportunity for improving patient outcomes [4]. Despite great advances in various imaging methods, the early diagnosis of PC still faces immense challenges at present.

Cancer-related biomarkers are defined as diagnostic indicators characterizing tumor biological behavior [5]. Among them, chemokine (C-X-C motif) receptor 4 (CXCR4) and integrin α_v_β_3_ are involved in several key biological processes of cancer and have been extensively investigated as molecular imaging targets. The overexpression of CXCR4 contributes to tumor growth, invasion, angiogenesis, metastasis, relapse, and drug resistance and is associated with an overall poor prognosis [6]. Furthermore, CXCR4 is involved in tumorigenesis and progression in many cancers, especially PC. CXCR4 is considered a key factor in the malignant transformation of pancreatic intraepithelial neoplasia, and its expression level may be related to the grade of neoplasia [7]. In addition, CXCR4 is involved in almost every aspect of PC, especially its invasion, metastasis, and chemoresistance [8]. Integrin α_v_β_3_ mediates interactions between cells and the extracellular matrix and has an important role in tumor angiogenesis [9,10]. The CXCL12/CXCR4 axis synergistically promotes angiogenesis by enhancing the production of vascular endothelial growth factor (VEGF) and matrix metalloproteinases (MMPs) as well as promoting integrin α_v_β_3_ expression [11]. Of note, CXCR4 and integrin α_v_β_3_ had a synergistic effect on cancer metastasis [12]. Therefore, molecular imaging probes targeting CXCR4 or integrin α_v_β_3_ hold great promise for PC.

Many molecular agents have been developed based on the structures of CXCR4 antagonists (such as plerixafor (AMD3100) [13], T140, FC131 [14], or antibodies [15]) or arginine-glycine-aspartate (RGD) derivatives for CXCR4 or integrin α_v_β_3_-targeted diagnosis and therapy [16,17,18,19]. Although promising results were achieved by these agents, mono receptor recognition may limit their sensitivity for the detection of diseases because of the heterogeneity of tumors and their relatively low receptor expression density, especially during the early stages [20,21]. Therefore, it is essential to develop radiotracers that bind to multiple receptors simultaneously, allowing tumor contrast when either receptor is expressed, thus improving the detection sensitivity. The development of heterodimeric tracers that bind to two different receptors with suitable pharmacokinetics and targeting efficiency is thought to be an effective solution [22,23].

Over the past five years, there has been a growing interest in the development and evaluation of tracers possessing heterobivalent targeting properties for tumor diagnosis and treatment. Biomarkers including integrin α_v_β_3_, GRPR (gastrin-releasing peptide receptor), MC1R (melanocortin receptor subtype 1), SSTRs (somatostatin receptors), PSMA (prostate-specific membrane antigen), VPAC_1_R (vasoactive intestinal peptide receptor subtype 1), NPY(Y_1_)R (neuropeptide Y receptor subtype 1) were extensively investigated as one of the targeting moieties [24,25,26,27,28,29,30,31,32,33], and the developed heterodimeric tracers demonstrating improved targeting affinity and efficiency compared with their corresponding single-receptor targeted tracers. Especially, BBN-RGD was further assessed in breast cancer and prostate cancer patients and exhibited promising value in diagnosis and staging [25,26]. As to the diagnosis of PC, NGR-RGD and BBN-RGD exhibited promising results in preclinical evaluations [26,34]. In addition, this heterodimeric strategy was also applied in the development of nanoparticle-based probes, displaying a unique advantage in improving tumor targeting efficiency [35].

The aim of this study was to develop a dual receptor targeting peptide heterodimer probe, which can target CXCR4 and integrin α_v_β_3_ simultaneously, to improve the detection sensitivity of tumors, especially PC. The in vitro and in vivo performance of the developed tracer ^68^Ga-yG5-RGD was investigated using a PC tumor model and the results were compared with their corresponding single-target probes, ^68^Ga-yG5 and ^68^Ga-RGD.

## 2. Materials and Methods

### 2.1. Reagents and Instruments

Gallium-68 was eluted from a commercial ^68^Ga/^68^Ge-generator (Isotope Technologies Garching GmbH, Garching, Germany) with 0.05 M HCl. Peptide c(RGDyK) was customized by Chinapeptide (Shanghai, China). Endo-BCN-PEG_4_-NHS ester was purchased from Broadpharm (San Diego, CA, USA). The 2,2′,2″-(2-(4-isothiocyanatobenzyl)-1,4,7-triazonane-1,4,7-triyl)triacetic acid (*p*-SCN-Bn-NOTA) was purchased from Macrocyclics (Dallas, TX, USA). NOTA-Bn-thioureido-cyclic(Arg-Gly-Asp-d-Tyr-Lys) (NOTA-c(RGDyK)), NO2A_tBu_-N_3_, and BCN-PEG_4_-c(RGDyK) were synthesized according to published methods [24]. All other chemicals were purchased from J&K Chemicals (Beijing, China), Adamas Reagent Co., Ltd., (Shanghai, China), or Sigma-Aldrich (St. Louis, MO, USA) unless indicated otherwise. The chemicals were used directly without further purification. Radioactivity of all samples was quantified by an automatic gamma counter (2470 WIZARD; PerkinElmer, Waltham, MA, USA). TransPET Discoverist 180 small animal PET/CT (Raycan Technology Co., Ltd., Suzhou, China) and lnliView-3000B small animal PET/SPECT/CT (Novel Medical, Beijing, China) were used to visualize mice with xenografted tumors. High-performance liquid chromatography (HPLC) was performed on LC-20AT (Shimadzu Corporation, Tokyo, Japan) equipped with an SPD-20A UV/VIS detector and a flow count radiation detector (Bioscan, Washington, DC, USA). Radiochemical yield and radiochemical purity of the tracers were determined by HPLC using a 4.6 × 250 mm Luna C_18_ column (Phenomenex, CA, USA). The column was eluted at a flow rate of 1 mL/min at 25 °C with a gradient of 5–90% MeCN/H_2_O with 0.1% trifluoroacetic acid (TFA) in 10 min.

### 2.2. Synthesis of NOTA-yG5 and NOTA-yG5-RGD

Cyclic peptide Cyclo(D-Tyr-N-Me-D-Lys-Arg-2-Nal-Gly), denoted as yG5, was an analogy of peptide FC131, designed as a new CXCR4 antagonist. The detailed evaluation of yG5 was published in an issued patent [36] and the results are considered published as an article elsewhere. Peptide yG5 was prepared using Fmoc-based solid phase peptide synthesis method and then conjugated with *p*-SCN-Bn-NOTA to obtain NOTA-yG5 after purification (Scheme S1). High-resolution mass spectrometry (HRMS) (Bruker SolariX 7.0T, Bruker Daltonik, Bremen, Germany): *m*/*z* calcd for C_57_H_75_N_13_O_12_S [M + 2H]^2+^ 583.7767, Found 583.7756. 

To construct CXCR4/integrin α_v_β_3_ heterodimeric ligand, bifunctional chelator NO2A_tBu_-N_3_ was applied to conjugate yG5 with c(RGDyK) using our previously developed method [23,26]. Briefly, NO2A_tBu_-N_3_ was first conjugated to peptide yG5 to obtain N_3_-NOTA-yG5 after TFA deprotection and HPLC purification. The heterodimeric tracer Cyclo(D-Tyr-N-Me-D-Lys-Arg-2-Nal-Gly)-NOTA-click-PEG_4_-c(RGDyK) (denoted as NOTA-yG5-RGD) was then obtained via copper-free click chemistry by reacting N_3_-NOTA-yG5 with BCN-PEG_4_-c(RGDyK) [24] at a molar ratio of 1:1 and purifying using HPLC (Scheme S1). High resolution mass spectrometry (HRMS) (Bruker SolariX 7.0T, Bruker Daltonik, Bremen, Germany): *m*/*z* calcd for C_101_H_146_N_24_O_26_: [M + 2H]^2+^ 1056.5499; found 1057.0540.

### 2.3. Radiolabeling

In brief, NOTA-yG5-RGD (4 nmol), NOTA-c(RGDyK) (4 nmol) or NOTA-yG5 (4 nmol) was separately added to 150 µL sodium acetate buffer (0.25 M). Subsequently, 500 μL of [^68^Ga]GaCl_3_ (0.05 M HCl, 296~370 MBq) was added, vortex mixing for 30 s. The final pH of the mixed solution was about 4.0. The reaction mixture was then heated to 100 °C for 5 min. The resultant products were analyzed by analytical radio-HPLC to determine the radiochemical yield and purity. 

### 2.4. Stability and Octanol/Water Partition Coefficient (logP)

The ^68^Ga-yG5-RGD (1.85 MBq, 5 μL) or ^68^Ga-yG5 (1.85 MBq, 5 μL) was separately added to 300 μL of PBS or normal human fresh serum, incubated for 2 h at 37 °C. After incubation, the PBS sample was subjected to HPLC for radiochemical purity under the conditions described above. An equal volume of anhydrous acetonitrile was added to serum sample, mixed, and then centrifuged at 13,000 g for 10 min to fully precipitate protein. Following this, the supernatant was collected and authenticated by HPLC.

Two normal BALB/c nude mice (female, 8 weeks) were used to evaluate in vivo metabolic stability of the two probes. The ^68^Ga-yG5-RGD or ^68^Ga-yG5 of about 74 MBq per mouse was intravenously injected via the tail vein. Two hours later, mice were anesthetized by intraperitoneal injection of 1% pentobarbital sodium solution (0.1 mL/20 g). Urine samples were collected by gentle bladder massage. After adding an equal volume of acetonitrile, the mixture was centrifuged, and the supernatant was analyzed by HPLC.

^68^Ga-yG5-RGD, ^68^Ga-yG5, or ^68^Ga-RGD (740 kBq each) were separately added to a mixture of 1.0 mL n-octanol and 1.0 mL PBS, and then the mixtures were thoroughly vortexed for 5 min. Each sample was then centrifuged at 8000 rpm for 4 min. Each sample (100 μL) in organic phase and aqueous phase was collected separately in tubes, 5 tubes for each phase. Radioactive counts per tube of samples were measured by gamma counter, and log*P* values were calculated by the following formula: log*P* = log10 (counts in n-octanol/counts in PBS).

### 2.5. Cell Culture

The BxPC3 human pancreatic cancer cell line was kindly provided by the Laboratory of Pancreatic Surgery at the Huazhong University of Science and Technology Affiliated Union Hospital (obtained from Shanghai Cell Bank of the Chinese Academy of Sciences, Shanghai, China). The MX-1 human breast cancer cell line was kindly provided by Basic Medical School of Huazhong University of Science and Technology. BxPC3 and MX-1 cells were cultured in DMEM containing 10% fetal bovine serum and 1% penicillin-streptomycin at 37 °C in a 5% CO_2_ incubator.

### 2.6. Western Blot Assay

Briefly, the BxPC3 and MX-1 cells were separately cultured in 6-well plates at the density of 2 × 10^5^ cells/well. After the cells adhered to the wall, the total protein was extracted. Protein concentrations were determined by BCA protein assay kit. After the preparation of 10% SDS-PAGE separation gel and concentrated gum, 40 μg total protein samples and marker were separately loaded into each well of the SDS-PAGE gel for separation by electrophoresis. Following electrophoresis, proteins were transferred onto PVDF membranes. The membrane was later incubated with anti-Integrin α_ν_, anti-Integrin β_3_, and anti-CXCR4 rabbit monoclonal polyclonal antibodies (1:1000 dilution) overnight at 4 °C. Incubation was for 1 h at room temperature with secondary antibody (1:20,000 dilution) and development was by enhanced chemiluminescence (ECL). The densities of the bands were quantified by Image J Software (version 1.45, NIH, Bethesda, MA, USA).

### 2.7. In Vitro Cell Assays

BxPC3 cells in the logarithmic growth phase were harvested, counted, and seeded onto 24-well plate with 2 × 10^5^ cells/well and cultured overnight. The original culture medium was discarded and fresh DMEM (serum and antibiotic-free) was then added to continue culturing for 0.5 h. Subsequently, each well was separately added ^68^Ga-yG5-RGD, ^68^Ga-yG5, or ^68^Ga-RGD (74 kBq/well, 50 μL) and incubated for 30 min, 1 h, and 2 h (three replicate wells/each time point). At the end of incubation, the supernatant and the cell lysate were separately collected and radioactivity was measured by gamma counter. The cell uptake was expressed as the percentage of total applied radioactivity. Cell uptake study experiment was also performed using MX-1 as a control group for ^68^Ga-yG5-RGD. The detailed experimental procedure was the same as above.

BxPC3 was used for cell blocking experiments. One group was taken as a control and added only ^68^Ga-yG5-RGD (74 kBq/well, 50 μL, 1 pmol). Other groups were incubated with the blocking agents (AMD3100, RGD, AMD3100 + RGD unlabeled, 1000 pmol/10 μL, and ^68^Ga-yG5-RGD (74 kBq/well, 50 μL). The rest of the procedures were carried out as described above. 

### 2.8. Animal Models

All animal studies were conducted in accordance with the guidelines of the Institutional Animal Care and Use Committee of Tongji Medical College of Huazhong University of Science and Technology (2021-S2875; 1 February 2021). Female BALB/c nude mice (5–6 weeks old) were purchased from Beijing HFK Bioscience Co., Ltd. (Beijing, China) and were raised in a specific pathogen-free barrier system. For subcutaneous tumor xenograft model, 1 × 10^7^ cells of BxPC3 or MX-1 in 100 μL PBS were injected into the right flanks of nude mice. Animal models were used for follow-up experiments when tumors reached 8~10 mm in diameter.

Written informed consent from all patients was obtained and the experiments were approved by the institutional review board of Union Hospital, Tongji Medical College, Huazhong University of Science and Technology (2021-0799-01; 2 November 2021). For the establishment of patient-derived xenograft (PDX) models, tumor tissues from pancreatic cancer patients were obtained via surgical resection. Collected tumor tissues were immediately placed in a sterile tube containing ice-chilled culture medium with 1% penicillin/streptomycin, 5 ug/mL tetracycline, and 10 ug/mL ciprofloxacin. Anesthetize the mouse by exposure to 2~2.5% vaporized isoflurane in 100% air using an induction chamber, then transfer it to the surgical table and place it in a prone position and maintain anesthesia with 1.5~2% vaporized isoflurane through the nose cone. The tumor tissues were then minced into 1~3 mm^3^ pieces and subcutaneously implanted into both flanks of BALB/c nude mice using a trocar. The PDX models were used for PET/CT scans when tumors reached a diameter of about 8~10 mm.

### 2.9. Small Animal PET/CT Static Scan

For comparison of in vivo performance of the probes, each group of BxPC3 tumor xenograft models (*n* = 4) was separately injected via tail vein with ^68^Ga-yG5-RGD, ^68^Ga-RGD, and ^68^Ga-yG5 (5.55~7.4 MBq, 150 μL/per mouse). Ten-minute static PET scan was acquired, followed by 6-min CT scan at 30 min, 1 h, and 2 h post-injection. Image data were reconstructed after attenuation correction. A region of interest (ROI) was drawn on the attenuation-corrected image to calculate the uptake value. In order to better assess the in vivo performance of ^68^Ga-yG5-RGD, pancreatic cancer-PDX models were also used for imaging. 

For testing of in vivo targeting specificity of ^68^Ga-yG5-RGD, nude mice bearing BxPC3 tumors (*n* = 4) were co-injected with ^68^Ga-yG5-RGD (5.55~7.4 MBq, 150 μL/per mouse) and blockers (AMD3100, RGD, or AMD3100 + RGD, 10 mg/kg each) via tail vein, and PET/CT scan was conducted at 30 min after injection. 

MX-1 tumor xenograft models (*n* = 4) were set as controls. Additionally, the detailed operation procedure was as described above. Briefly, nude mice bearing MX-1 tumor were injected with ^68^Ga-yG5-RGD (5.55~7.4 MBq, 150 μL/per mouse) and PET/CT scan was conducted at 30 min, 1 h, and 2 h after injection.

### 2.10. Biodistribution Studies

The group setting was kept consistent with PET/CT scan. Each group of mice (*n* = 4) was injected via tail vein with probes (5.55~7.4 MBq, 150 μL) or co-injected with probes and blockers (10 mg/kg). Mice were euthanized by cervical dislocation under anesthesia 2 h post-injection. Subsequently, tissues of interest were collected, washed, weighed, and counted by a gamma counter. After decay correction, uptake of tissues was expressed as the percentage of injected dose per gram of tissue (%ID/g).

### 2.11. Immunohistochemical Analysis

BxPC3, MX-1, and PDX tumor samples were collected, fixed, paraffin-embedded, and then cut into 5 µm sections. After antigen repair, endogenous peroxidase activity was blocked using a solution of 2.5% hydrogen peroxide in methanol. The tissue sections were incubated with 10% normal goat serum to reduce nonspecific binding. Incubate sections with primary antibody (anti-CXCR4, diluted 1:500; anti-α_v_β_3_, diluted 1:100; anti-CD31 1:100) overnight at 4 °C. The next day, the sections were incubated with a universal protein block (DAKO) for 25 min at room temperature. Sections were visualized with DAB (Dako) and observed under a light microscope. Subsequently, the sections were counterstained with hematoxylin. Additionally, the sections were dehydrated with gradient ethanol, sealed with neutral gum, and then detected under the microscope.

### 2.12. Statistical Analysis

All quantitative data are expressed as a mean ± standard deviation. Data were plotted and statistically analyzed using GraphPad Prism software (version 8.0; GraphPad Software Inc., La Jolla, CA, USA). Comparisons between groups were performed using Student’s *t*-test for independent samples. *p* < 0.05 was considered indicative of a significant difference.

## 3. Results

### 3.1. Chemical and Radiochemical Characterization

**Figure 1:**
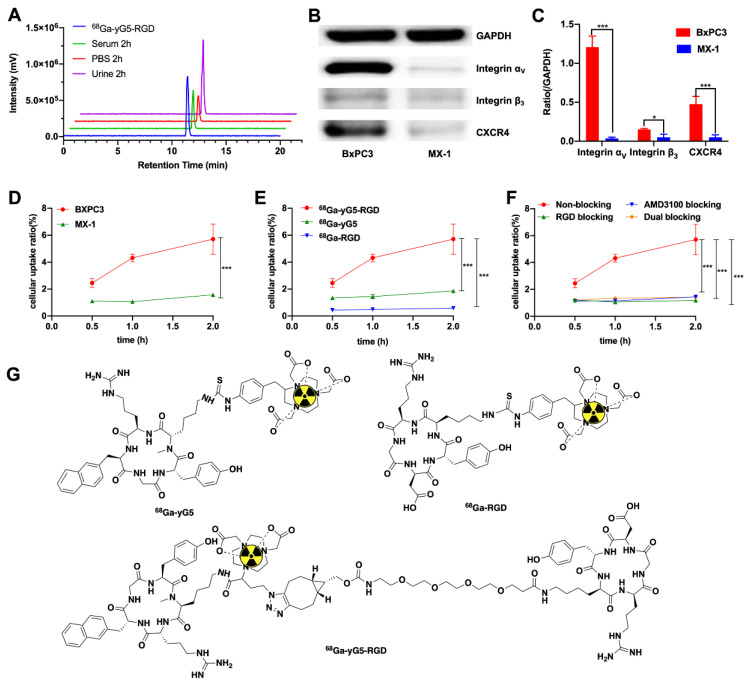
In vitro studies. (**A**) Identification and stability study of ^68^Ga-yG5-RGD. Analytic radio-HPLC chromatograms of ^68^Ga-yG5-RGD (blue). The 2 h in vitro stability of ^68^Ga-yG5-RGD in PBS (red), and serum (green). The 2 h in vivo stability of ^68^Ga-yG5-RGD in urine (purple). (**B**) Western blot was performed to evaluate the expression levels of CXCR4, integrin α_ν,_ and integrin β_3_ in BxPC3 and MX-1 cells, with GAPDH used as the internal control. (**C**) The semi-quantitative analysis was conducted through the integrated optical density ratio of CXCR4, integrin α_ν_, and integrin β_3_ to GADPH (Data represent one of three separate experiments. mean ± SD). (**D**) The uptake of ^68^Ga-yG5-RGD in BxPC3 and MX-1 cells. (**E**) The uptake of ^68^Ga-yG5-RGD, ^68^Ga-yG5, and ^68^Ga-RGD by BxPC3 cells at 30 min, 1 h, and 2 h. (**F**) The uptake of ^68^Ga-yG5-RGD by BxPC3 cells was blocked by unlabeled RGD, AMD3100, and RGD + AMD3100. Three duplicate wells were set in each group. Data are expressed as mean ± SD, *n* = 3; * *p* <0.05, *** *p* < 0.001. (**G**) Chemical structures of ^68^Ga-yG5-RGD, ^68^Ga-yG5, and ^68^Ga-RGD.

The heterodimeric agent NOTA-yG5-RGD was conveniently prepared via a click chemistry-based method as described in the Methods section (Scheme S1). The as-prepared NOTA-yG5 and NOTA-yG5-RGD were characterized and identified using high-resolution mass spectrometry and analytical HPLC before further evaluation. The mass spectra are shown in Figure S1. The radiolabeling process of the precursors was straightforward with radiochemical yields greater than 99% and 95% for ^68^Ga-yG5-RGD and ^68^Ga-yG5, respectively (Figure 1A). The molar activities of the resulting tracers were in the range of 74–92 MBq/nmol.

### 3.2. Stability and log P of ^68^Ga-yG5-RGD

The tracers were incubated in fresh human serum and PBS for 2 h at 37 °C and then analyzed by radio-HPLC. As shown in Figure 1 and Figure S2, the proportion of intact ^68^Ga-yG5-RGD was greater than 99%, and less than 5% of disassociated ^68^Ga was observed in ^68^Ga-yG5 samples, indicating well in vitro stability. BALB/c nude mice were used to evaluate the in vivo metabolic stability of ^68^Ga-yG5-RGD and ^68^Ga-yG5. Almost no degradation of ^68^Ga-yG5-RGD was observed in urine samples at 2 h post-injection (p.i.) (Figure 1A). The ^68^Ga-yG5 underwent some metabolism (~10%) in the urine samples (Figure S2). The log *p* values of ^68^Ga-yG5-RGD, ^68^Ga-RGD, and ^68^Ga-yG5 were −2.47 ± 0.04, −3.22 ± 0.02, and −0.35 ± 0.09 separately.

### 3.3. Western Blot Analysis

Target protein expression was analyzed by Western blot. BxPC3 cells had a relatively high expression of integrin α_ν_, moderate expression of CXCR4, and low expression of integrin β_3_, whereas MX-1 cells had low or no expression of CXCR4, integrin α_ν_, and β_3_ (Figure 1B,C).

### 3.4. In Vitro Cell Assays

Cellular uptake studies of ^68^Ga-yG5-RGD, ^68^Ga-RGD, and ^68^Ga-yG5 were performed using BxPC3 cells. All tracers showed an increase in uptake with prolonged incubation time (Figure 1E). The cellular uptake of ^68^Ga-yG5-RGD in BxPC3 cells was significantly higher than that of ^68^Ga-RGD and ^68^Ga-yG5 at all time points investigated (all *p* < 0.001).

The targeting specificity of ^68^Ga-yG5-RGD was evaluated by cellular blocking experiments (Figure 1F). When co-incubated with a 1000-fold molar excess amount of unlabeled RGD, AMD3100, or AMD3100 + RGD, the cellular uptake of ^68^Ga-yG5-RGD in BxPC3 cells was significantly decreased (all *p* < 0.001). The cellular uptake of ^68^Ga-yG5-RGD in the negative control MX-1 cells was also significantly lower than that of BxPC3 cells at all time points investigated (all *p* < 0.001, Figure 1D).

### 3.5. Small Animal PET/CT Scans

**Figure 2:**
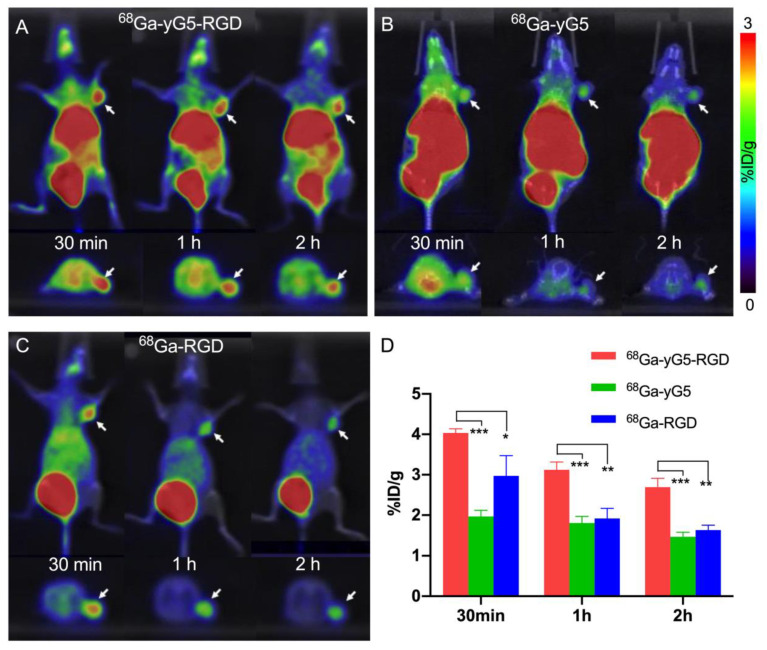
PET/CT scan of ^68^Ga-yG5-RGD in BxPC3 tumor-bearing mice. (**A**) Representative static PET/CT images of BxPC3 xenograft tumor mice at 30 min, 1 h, and 2 h p.i. of ^68^Ga-yG5-RGD, (**B**) ^68^Ga-yG5, and (**C**) ^68^Ga-RGD (each 5.55–7.4 MBq). (**D**) Tumor uptakes determined by quantitative ROI analysis of PET/CT images. Arrows indicate the location of tumors, *n* = 4; * *p* < 0.05, ** *p* < 0.01, *** *p* < 0.001.

To compare the in vivo targeting efficiency of mono- and dual-receptor targeting tracers, PET/CT scans were performed using BxPC3 tumor-bearing nude mice. PET images were acquired at 30 min, 1 h, and 2 h p.i. of the tracer. As shown in Figure 2A, ^68^Ga-yG5-RGD was mainly distributed in the abdomen (liver and kidneys) and bladder, and was excreted via the liver, gastrointestinal tract, and kidneys. BxPC3 tumors were clearly visualized at 30 min p.i. of ^68^Ga-yG5-RGD. The tumor outline was still very clear at 1 h and 2 h after injection. The metabolic pathway of ^68^Ga-yG5 was similar to that of ^68^Ga-yG5-RGD, with high radioactivity accumulation in the liver, gastrointestinal tract, and kidneys. The outline of tumors was clearly distinguished in the PET images, although high radioactivity was distributed throughout the body (Figure 2B). Regarding ^68^Ga-RGD, high radioactivity accumulation was found in the bladder and kidneys, as well as the liver (Figure 2C). The tumor was clearly visible early after administration by ^68^Ga-RGD and became fainter over time. The quantitation of the tracer accumulation of tumors on PET images was performed by drawing a region of interest (ROI) on the coronal images. The mean tumor uptake of the three tracers in BxPC3 tumors is shown in Figure 2D. The tumor uptake of ^68^Ga-yG5-RGD was substantially higher than that of ^68^Ga-RGD and ^68^Ga-yG5.

**Figure 3:**
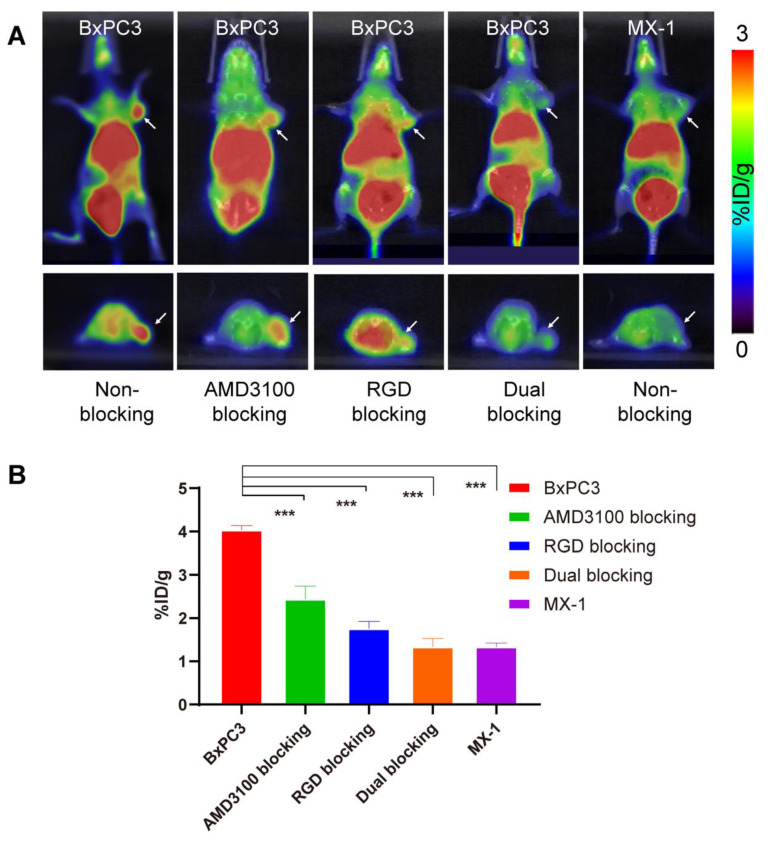
PET imaging and quantitative analysis show the specificity of ^68^Ga-yG5-RGD for CXCR4 and integrin α_v_β_3_ expression on tumors. (**A**) Decay-corrected whole-body small animal PET/CT images of BxPC3 or MX-1 tumor-bearing mice at 30 min after injection of 5.55–7.4 MBq of ^68^Ga-yG5-RGD and a blocking dose of AMD3100 (10 mg/kg), RGD (10 mg/kg), or AMD3100 (10 mg/kg) + RGD (10 mg/kg). (**B**) Tumor uptakes determined by quantitative ROI analysis of PET/CT images. Arrows indicate the location of tumors, *n* = 4; *** *p* < 0.001.

Several blocking assays were conducted to evaluate the in vivo binding specificity of ^68^Ga-yG5-RGD towards CXCR4 and integrin α_v_β_3_. As shown in Figure 3, the tumor uptake of ^68^Ga-yG5-RGD was substantially reduced when ^68^Ga-yG5-RGD was co-injected with the blockers AMD3100 (10 mg/kg), RGD (10 mg/kg), or AMD3100 (10 mg/kg) + RGD (10 mg/kg). Of note, images of the tumors in the dual-blocking groups were much fainter than those in the mono-blocking groups.

As a control, the in vivo performance of ^68^Ga-yG5-RGD was studied in MX-1 tumor-bearing mice with a low expression of CXCR4 and integrin α_v_β_3_ (Figure 3A). Low uptake of ^68^Ga-yG5-RGD was observed in MX-1 tumors with an intensity similar to that of the opposite shoulder of the mice. A quantitative ROI analysis of MX-1 tumors is shown in Figure 3B. The uptake of ^68^Ga-yG5-RGD by MX-1 tumors was 1.42 ± 0.16 %ID/g (*p* < 0.001) at 30 min, which was significantly lower than that in BxPC3 tumors.

### 3.6. Biodistribution Study

**Figure 4:**
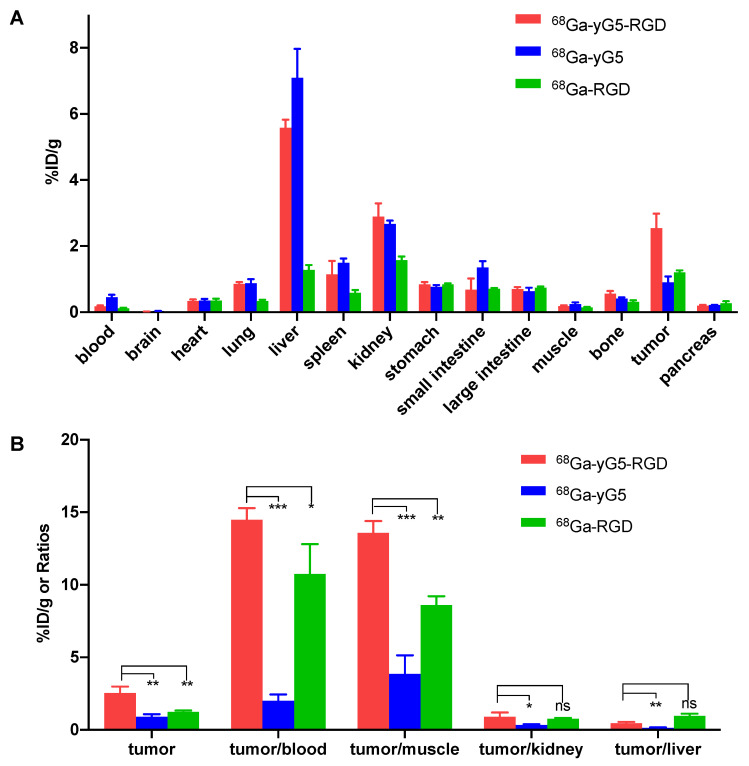
Biodistribution studies of the tracers. (**A**) Biodistribution studies of ^68^Ga-yG5-RGD, ^68^Ga-yG5, and ^68^Ga-RGD in BxPC3 xenograft mice at 2 h p.i. (each 5.55–7.4 MBq). (**B**) Analysis of tumor-to-blood, tumor-to-muscle, tumor-to-kidney, and tumor-to-liver ratios. Data are expressed as mean ± SD, *n* = 4; ns: not significant, * *p* < 0.05, ** *p* < 0.01, *** *p* < 0.001.

The in vivo distribution of the three tracers was investigated via a biodistribution study. As shown in Figure 4 and Table S1, ^68^Ga-yG5-RGD and ^68^Ga-yG5 showed the highest accumulation in the liver, followed by the kidney, demonstrating their metabolism and excretory pathway occurred in the liver and kidneys. The highest uptake of ^68^Ga-RGD was in the kidney, followed by the liver, demonstrating a different metabolic pathway. The tumor uptake of ^68^Ga-yG5-RGD (2.54 ± 0.44 %ID/g) was significantly higher than that of ^68^Ga-yG5 (0.90 ± 0.18 %ID/g, *p* < 0.01) and ^68^Ga-RGD (1.21 ± 0.06 %ID/g, *p* < 0.01). Since the tumor-to-nontumor ratios provide important information, especially for the choice of indications in the future clinical translation of the tracers, several tumor-to-nontumor ratios were calculated and compared. The tumor-to-blood (14.48 ± 0.80 vs. 2.01 ± 0.43, *p* < 0.001), tumor-to-muscle (13.58 ± 0.81 vs. 3.86 ± 1.29, *p* < 0.001), tumor-to-liver (0.90 ± 0.29 vs. 0.34 ± 0.06, *p* < 0.05), and tumor-to-kidney (0.46 ± 0.10 vs. 0.13 ± 0.04, *p* < 0.05) ratios of ^68^Ga-yG5-RGD were higher than those of ^68^Ga-yG5. The tumor-to-blood, tumor-to-muscle, and tumor-to-kidney ratios of ^68^Ga-yG5-RGD were higher than those of ^68^Ga-RGD (10.76 ± 2.04, *p* < 0.05; 8.61 ± 0.61, *p* < 0.01; 0.77 ± 0.06, *p* > 0.05, respectively). However, the tumor-to-liver ratio of ^68^Ga-yG5-RGD was slightly lower than that of ^68^Ga-RGD (0.96 ± 0.15, *p* > 0.05).

**Figure 5:**
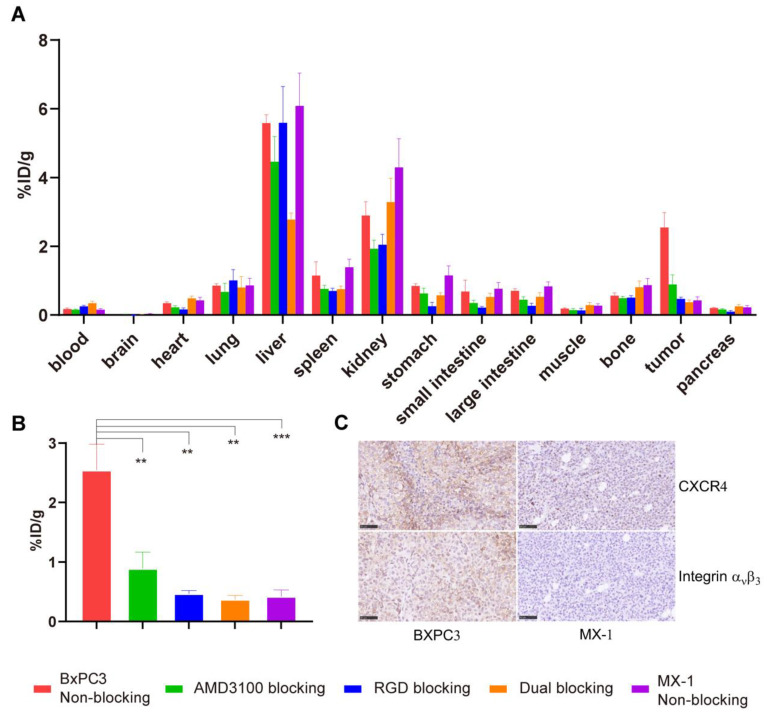
Ex vivo biodistribution studies and immunohistochemistry assays show the specificity of ^68^Ga-yG5-RGD for CXCR4 and integrin α_v_β_3_ expression on tumors. (**A**,**B**) Biodistribution results and tumor uptake comparisons of ^68^Ga-yG5-RGD in BxPC3 tumors with or without co-injection of excess amount of unlabeled AMD3100 (10 mg/kg), RGD (10 mg/kg), and AMD3100 (10 mg/kg) + RGD (10 mg/kg) and in MX-1 tumors at 2 h p.i. (each 5.55–7.4 MBq). (**C**) Immunohistochemistry assay of CXCR4 and integrin α_v_β_3_ in BxPC3 and MX-1 tumors (×400). Data are expressed as mean ± SD, *n* = 4; ** *p* < 0.01, *** *p* < 0.001, Scale bar = 50 μm.

To validate the in vivo targeting specificity of ^68^Ga-yG5-RGD, blocking biodistribution assays were conducted in BxPC3 xenograft mice. Each mouse was intravenously injected with ^68^Ga-yG5-RGD alone or co-injected with AMD3100 (10 mg/kg), RGD (10 mg/kg), or AMD3100 (10 mg/kg) + RGD (10 mg/kg). Mice were sacrificed at 2 h p.i., and ex vivo biodistribution analyses were performed. BxPC3 tumor uptake of ^68^Ga-yG5-RGD in the blocked groups (AMD3100, 0.89 ± 0.28 %ID/g, *p* < 0.001; RGD, 0.47 ± 0.05 %ID/g, *p* < 0.001; AMD3100 + RGD, 0.37 ± 0.07 %ID/g, *p* < 0.001) was significantly reduced compared with the non-blocked groups (2.54 ± 0.44 %ID/g) (Figure 5 and Table S2). 

A biodistribution study was also performed in MX-1 xenograft mice as a negative control model. At 2 h p.i., the MX-1 tumor uptake was 0.42 ± 0.11 %ID/g, which was remarkably lower than that in the BxPC3 tumor model (Figure 5 and Table S3, *p* < 0.001).

### 3.7. PET Imaging of Pancreatic Cancer PDX Model

**Figure 6:**
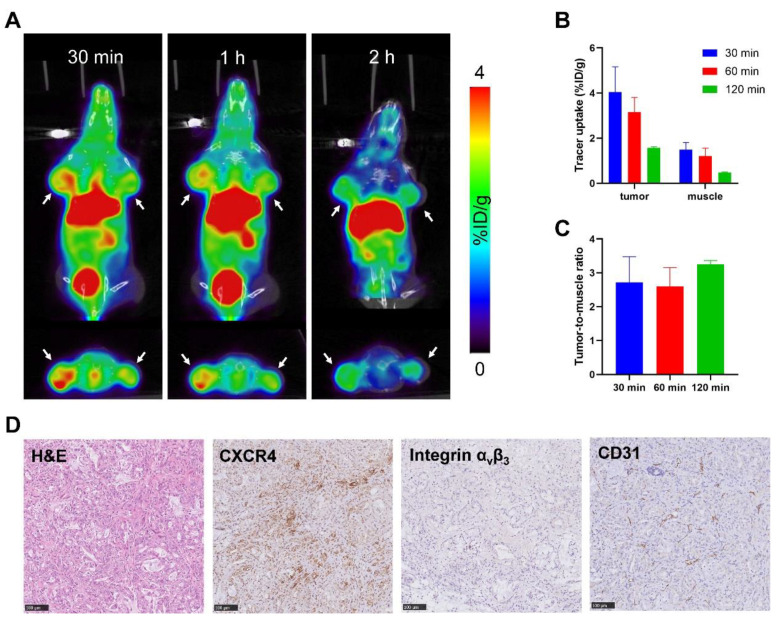
PET/CT scan and immunohistochemistry assay in pancreatic cancer-PDX models. (**A**) Representative static PET/CT images of pancreatic cancer-PDX models at 30 min, 1 h, and 2 h p.i. of ^68^Ga-yG5-RGD (each 5.55–7.4 MBq). Arrows indicate the location of tumors, *n* = 4. (**B**) Tracer uptakes in tumors and muscles determined by quantitative ROI analysis of PET/CT images. (**C**) Tumor-to-muscle ratios. (**D**) Immunohistochemistry assay of CXCR4, integrin α_v_β_3_ and CD31 in PDX-tumors (×200). Scale bar = 100 μm.

To further validate the utility of the developed tracer in PET imaging of pancreatic cancer and its potential for future translation, pancreatic cancer patient-derived xenograft (PDX) models were developed and PET imaging using ^68^Ga-yG5-RGD was applied after the xenografts reached 8~10 mm in diameter. The overall biodistribution profiles were similar to the results of BxPC3 cell-derived xenografts. For example, all the tumors in PDX models were clearly visualized from 30 min to 2 h after injection of ^68^Ga-yG5-RGD (Figure 6). Higher uptake was observed in relatively larger tumors compared to smaller tumors, which may be due to the maturing of the blood vascular system. The tumor uptake was 4.05 ± 1.12 %ID/g, 3.15 ± 0.66 %ID/g, and 1.57 ± 0.05 %ID/g at 30 min, 1 h, and 2 h post-injection of the tracer, which are very close to the uptake values of BxPC3 xenografts. 

### 3.8. Immunohistochemical Staining

Expressions of CXCR4 and integrin α_v_β_3_ in tumor tissues were analyzed by immunohistochemistry. As shown in Figure 5C, the BxPC3 tumors exhibited relatively high expressions of CXCR4 and integrin α_v_β_3_, whereas low or no expression of CXCR4 and integrin α_v_β_3_ proteins were found in MX-1 tumors. The immunohistochemistry results were consistent with the data from the Western blot analysis. As for PDX models (Figure 6D), tumors showed high CXCR4 expression, moderate α_v_β_3_, and CD31 expression.

## 4. Discussion

Recently, the use of radiotracers targeting CXCR4 or integrin α_v_β_3_ for tumor imaging has been widely investigated [17,18]. However, monomeric peptides usually accumulate in tumors with a relatively low concentration because of their moderate affinity for their targets and the heterogeneity of tumors, resulting in unsatisfied tumor detection sensitivity [22]. Therefore, to improve the sensitivity of tracers for tumor imaging, we used a polyvalent strategy to develop a heterodimeric tracer, ^68^Ga-yG5-RGD, which targeted CXCR4 and integrin α_v_β_3_ simultaneously for PC imaging. Compared with monospecific tracers, the dual-receptor targeting tracer ^68^Ga-yG5-RGD had enhanced tumor-targeting efficiency and prolonged tumor retention, suggesting their high clinical translation potential.

The preparation and radiolabeling process for ^68^Ga-yG5-RGD was simple and achieved a high radiochemical yield and purity, and there was no requirement for extra purification steps. The ^68^Ga-yG5-RGD also exhibited excellent in vitro and in vivo stability, with less than 1% metabolic-free Gallium-68 observed. It should be noticed that a shoulder peak was found in the main peak of the 2 h urine sample, which might be due to the metabolism of the tracer or the increased tube circles that pass through the detector cell to improve the detecting sensitivity of the gamma detector. The cellular uptake of ^68^Ga-yG5-RGD was considerably higher than that of ^68^Ga-yG5 or ^68^Ga-RGD in BxPC3 cells that express CXCR4 and integrin α_v_β_3_, demonstrating the higher target efficiency of dual-targeted tracers than single-targeted tracers. The uptake of ^68^Ga-yG5-RGD in BxPC3 cells was notably blocked by an excess of unlabeled AMD3100, RGD, or AMD3100 + RGD, suggesting a favorable targeting specificity towards the two receptors, CXCR4, and integrin α_v_β_3_.

The in vivo performance of ^68^Ga-yG5-RGD was evaluated by small animal PET/CT in BxPC3 tumor-bearing nude mice as well as PDX models. Within the observation time, high levels of radioactivity were observed in the liver and kidneys after the injection of ^68^Ga-yG5-RGD, which indicated ^68^Ga-yG5-RGD was metabolized and excreted mainly via the liver and kidneys. The outline of the BxPC3 tumor was more clearly visualized by ^68^Ga-yG5-RGD compared with ^68^Ga-yG5 or ^68^Ga-RGD. The ROI analysis showed that the uptake of ^68^Ga-yG5-RGD by BxPC3 tumors was markedly higher than that of ^68^Ga-yG5 or ^68^Ga-RGD at all time points studied. The quantitative biodistribution studies showed that the uptake of ^68^Ga-yG5-RGD by BxPC3 tumors was statistically higher than that of its monomeric counterparts, which was in concordance with the imaging results. Moreover, the tumor-to-blood and tumor-to-muscle ratios of ^68^Ga-yG5-RGD were higher than those of ^68^Ga-yG5 and ^68^Ga-RGD. This improved tumor-targeting efficiency might be attributed to the dual CXCR4 and integrin α_v_β_3_ binding ability of ^68^Ga-yG5-RGD in BxPC3 tumors. The increased molecular weight of the peptide heterodimer and the introduction of short polyethylene glycol groups may result in its prolonged blood circulation, which may also contribute to its enhanced tumor uptake. Similarly, ^68^Ga-yG5-RGD also showed well in vivo tumor targeting performance in PDX-tumors. It should be noticed that the RGD unit contributes a major role in improving the tumor-to-nontumor ratios, and the yG5 unit improved the tumor uptake values and prolonged the retention in the tumor. A synergistic effect may be expected when designing and developing a heterodimeric tracer.

To evaluate the in vivo dual-receptor targeting specificity of ^68^Ga-yG5-RGD, blocking studies were performed in BxPC3 tumor-bearing nude mice. A substantial reduction of radioactivity was observed in BxPC3 tumors when they were blocked by excess amounts of unlabeled AMD3100 or RGD, indicating the specific binding between ^68^Ga-yG5-RGD and its targets. Of note, the lowest uptake of ^68^Ga-yG5-RGD by BxPC3 tumors was observed when it was administered intravenously with the co-injection of AMD3100 and RGD. Interestingly, the yG5 motif of ^68^Ga-yG5-RGD could still bind to CXCR4 when blocked by RGD, and the RGD motif could still bind to integrin α_v_β_3_ when blocked by AMD3100. These blocking results suggested that ^68^Ga-yG5-RGD achieved dual-receptor targeting specificity. In addition, when compared with the control groups, the uptake of ^68^Ga-yG5-RGD by MX-1 tumors was similar to or even lower than that of muscles, further confirming its targeting specificity. Immunohistochemical analysis also confirmed that the levels of the receptors expressed on BxPC3, and MX-1 tumors were consistent with the uptake of the tracers.

Although promising results were achieved by this heterodimeric tracer, it should be noted that the accumulation of radioactivity in the liver was relatively high, which is unfavorable for detecting primary lesions of PC. The tumor-to-liver ratios of ^68^Ga-yG5-RGD and ^68^Ga-yG5 were relatively lower than that of ^68^Ga-RGD (no statistical difference), which might be related to the strong hydrophobic menaphthyl group and the positive charge of the arginine amino acid in the yG5 sequence, as well as better in vivo pharmacokinetic characteristics of RGD probes. In addition, normal liver tissues express multiple chemokine receptors, including CXCR4 [17], which might also explain the high accumulation of radioactivity related to ^68^Ga-yG5-RGD and ^68^Ga-yG5 in the liver. Compared with ^68^Ga-yG5, the hydrophilicity of ^68^Ga-yG5-RGD was markedly improved via PEGylation and the introduction of an RGD moiety, assessed by their log *p* values and the decreased intestinal uptake and hepatobiliary excretion of ^68^Ga-yG5-RGD in vivo. However, further modification of the structure of ^68^Ga-yG5-RGD is still needed to improve its in vivo pharmacokinetics, including extending the PEGylation and modifying the structure of the CXCR4 antagonist [37,38]. Another potential solution would be to pre-inject a small amount of CXCR4 antagonist to reduce liver uptake, thus improving tumor uptake [39]. Last but not least, the cell line BxPC3 used in the study showed moderate expression of both CXCR4 and integrin α_v_β_3_, which accounted for a part of the reason for suboptimal tumor imaging. For further experiments after the chemical improvement of the radiotracers, we will establish cell lines with high expression of CXCR4 and integrin α_v_β_3_ by stable transfection to better evaluate the performance of the tracers for pancreatic cancer imaging. Meanwhile, cell lines with low, moderate, and high expression of targets will be used to better evaluate the specificity of the heterodimer for the different targets.

Studies have reported that CXCR4 and integrin α_v_β_3_ are synergistically involved in cancer metastasis [12,40]. Therefore, ^68^Ga-yG5-RGD may have prospective applications in the detection of tumor metastatic foci. Moreover, although the BxPC3 cell line selected in our study has a moderate expression of CXCR4 and integrin α_v_β_3_, BxPC3 tumors could still be visualized clearly by ^68^Ga-yG5-RGD because of its high tumor uptake. Many human cancers have high expressions of CXCR4 and integrin α_v_β_3_ [6,11], suggesting ^68^Ga-yG5-RGD may be useful for a broad range of applications and have good prospects for clinical translation. Furthermore, with improved in vivo pharmacokinetics of this heterodimeric tracer, yG5-RGD could also be labeled by therapeutic radionuclides, such as ^177^Lu, providing an alternative method for the treatment of PC.

## 5. Conclusions

In this study, a peptide-based heterodimeric tracer ^68^Ga-yG5-RGD targeting CXCR4 and integrin α_v_β_3_ simultaneously was successfully developed with excellent in vitro and in vivo performance. This dual-receptor targeting strategy achieved improved tumor-targeting efficiency and prolonged tumor retention in BxPC3 tumors. Therefore, ^68^Ga-yG5-RGD is a promising probe for the noninvasive detection of tumors that express either CXCR4 or integrin α_v_β_3_ or both, indicating good prospects for clinical translation.

## Data Availability

The data presented in this study are available on request to the corresponding author.

## Supplementary Materials

The following supporting information can be downloaded at: https://www.mdpi.com/article/10.3390/pharmaceutics14091791/s1. Scheme S1. Synthesis route of NOTA-yG5 and NOTA-yG5-RGD; Figure S1. (A) Mass spectra of NOTA-yG5 and (B) NOTA-yG5-RGD; Figure S2. Identification and stability study of ^68^Ga-yG5; Table S1. Biodistribution results of ^68^Ga-yG5-RGD, ^68^Ga-yG5, and ^68^Ga-RGD in BxPC3 xenograft mice at 2 h p.i; Table S2. Biodistribution results of in vivo blocking assays; Table S3. Biodistribution results of ^68^Ga-yG5-RGD in BxPC3 and MX-1 xenograft mice at 2 h p.i.
