# Supplementary material for: Development and Evaluation of a Peptide Heterodimeric Tracer Targeting CXCR4 and Integrin αvβ3 for Pancreatic Cancer Imaging
Source: Pharmaceutics. 2022 Aug 26;14(9):1791. doi: 10.3390/pharmaceutics14091791 (PMC9503769; doi:10.3390/pharmaceutics14091791)
Supplement: Supplementary file 1 [file pharmaceutics-14-01791-s001.zip › pharmaceutics-1840115-supplementary.pdf]

Supplementary materials

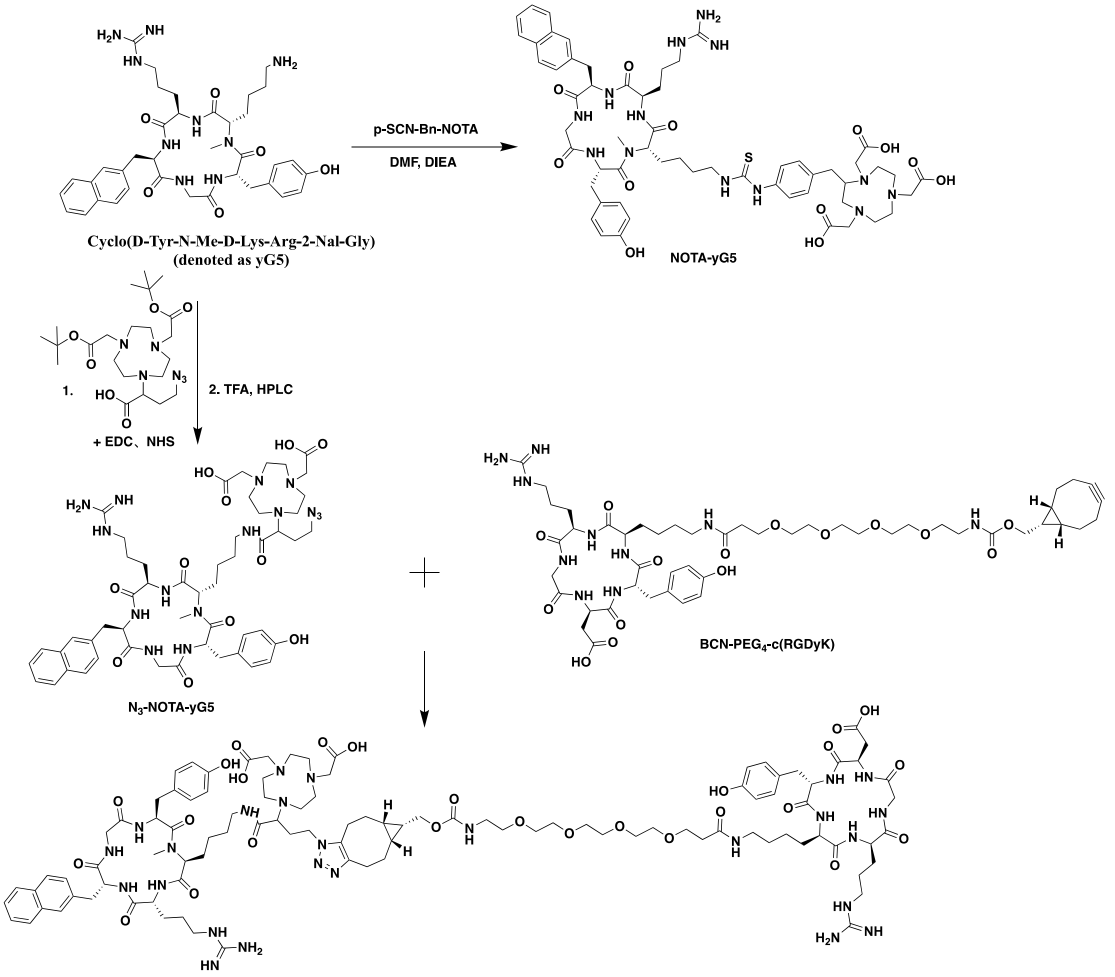

Scheme S1. Synthesis route of NOTA-yG5 and NOTA-yG5-RGD.

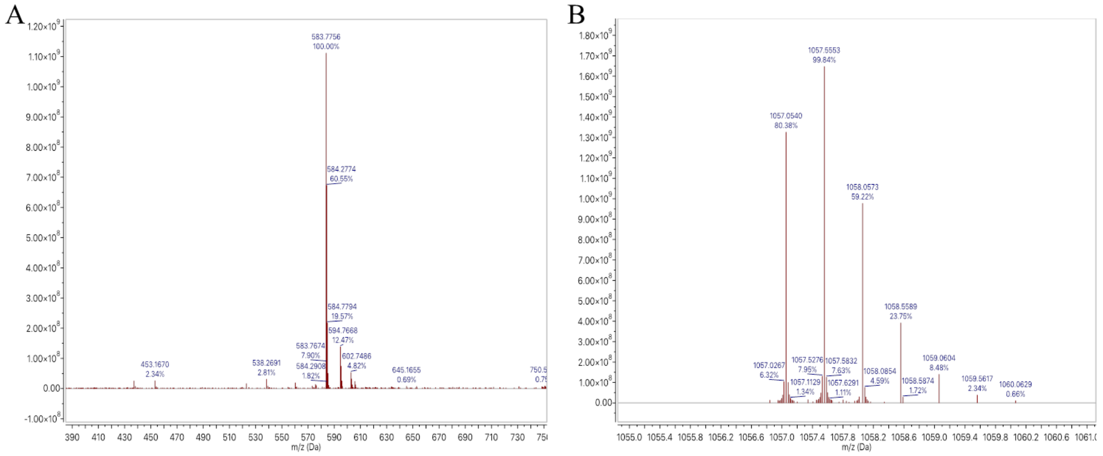

Figure S1. Mass spectra of NOTA-yG5 (A) and NOTA-yG5-RGD (B).

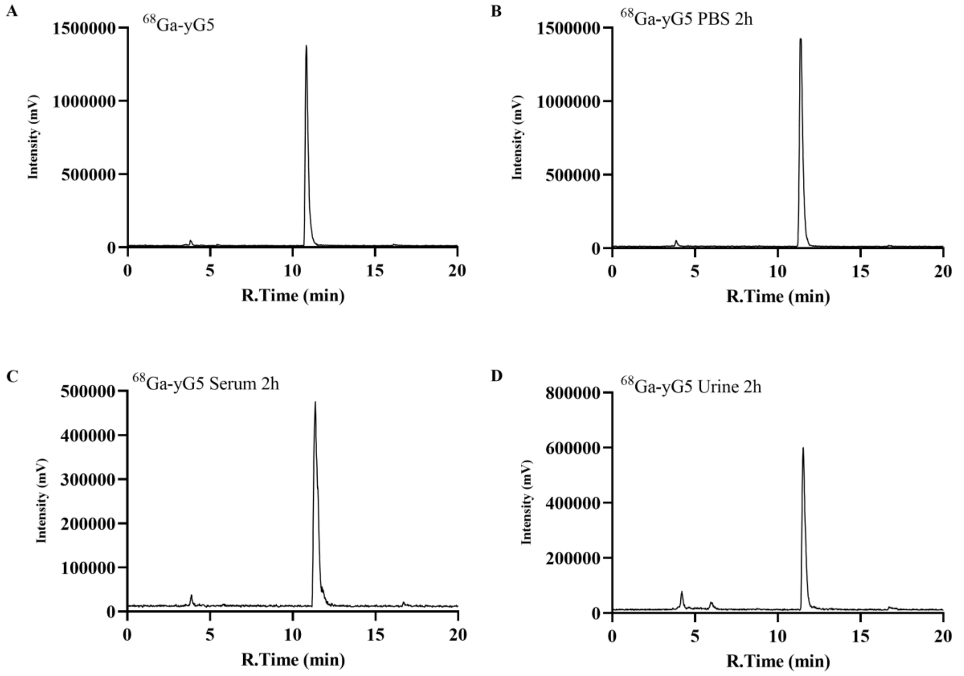

Figure S2. Identification and stability study of ^{68}Ga-yG5. Analytic radio-HPLC chromatograms of ^{68}Ga-yG5 (A). 2 h *in vitro* stability of ^{68}Ga-yG5 in PBS (B), and serum (C). 2 h *in vivo* stability of ^{68}Ga-yG5 in urine (D).

Table S1. Biodistribution results of ^{68}Ga-yG5-RGD, ^{68}Ga-yG5 and ^{68}Ga-RGD in BxPC3 xenograft mice at 90 min p.i.

organs / probes	^{68}Ga-yG5-RGD	^{68}Ga-yG5	^{68}Ga-RGD
blood	0.18±0.03	0.45±0.07	0.11±0.02
brain	0.02±0.00	0.03±0.00	0.02±0.00
heart	0.34±0.04	0.35±0.05	0.35±0.06
lung	0.85±0.06	0.87±0.12	0.34±0.03
liver	5.58±0.24	7.08±0.88	1.27±0.15
spleen	1.15±0.41	1.49±0.13	0.59±0.08
kidney	2.89±0.40	2.67±0.10	1.58±0.11
stomach	0.85±0.07	0.76±0.06	0.84±0.04
small intestine	0.68±0.34	1.35±0.19	0.71±0.03
large intestine	0.70±0.06	0.63±0.10	0.74±0.04
muscle	0.19±0.02	0.24±0.05	0.14±0.02

bone	0.56±0.08	0.41±0.04	0.31±0.06
tumor	2.54±0.44	0.90±0.18**	1.21±0.06**
pancreas	0.20±0.02	0.21±0.00	0.27±0.07
**uptake ratio**			
tumor/blood	14.48±0.80	2.01±0.43***	10.76±2.04*
tumor/muscle	13.58±0.81	3.86±1.29***	8.61±0.61**
tumor/kidney	0.90±0.29	0.34±0.06*	0.77±0.06ns
tumor/liver	0.46±0.10	0.13±0.04*	0.96±0.15 ns

(Note: each 5.55–7.4 MBq. Data are presented as mean ± SD (%ID/g, n = 4). ns P>0.05, *P<0.05, **P<0.01, ***P<0.001)

Table S2. Biodistribution results of *in vivo* blocking assays.

organs / blocking	AMD3100 blocking	RGD blocking	Dual blocking
blood	0.15±0.02	0.25±0.03	0.34±0.06
brain	0.02±0.01	0.01±0.00	0.02±0.00
heart	0.22±0.05	0.16±0.05	0.48±0.07
lung	0.67±0.25	1.01±0.31	0.80±0.32
liver	4.46±0.73	5.59±1.06	2.78±0.19
spleen	0.76±0.10	0.70±0.08	0.75±0.09
kidney	1.93±0.25	2.04±0.31	3.29±0.69
stomach	0.63±0.15	0.26±0.11	0.57±0.08
small intestine	0.35±0.08	0.22±0.03	0.52±0.11
large intestine	0.45±0.08	0.27±0.08	0.53±0.12
muscle	0.14±0.05	0.14±0.06	0.28±0.08
bone	0.49±0.05	0.50±0.06	0.81±0.18
tumor	0.89±0.28**	0.47±0.05**	0.37±0.07**
pancreas	0.16±0.03	0.10±0.03	0.25±0.06

(Note: Each mouse was co-injected of excess amount of unlabeled AMD3100 (10mg/kg), RGD (10mg/kg), and AMD3100 (10mg/kg) + RGD (10mg/kg) at 30 min p.i. each 5.55–7.4 MBq. Data

are presented as mean ± SD (%ID/g, n = 4). ns P>0.05, **P<0.01)

Table S3. Biodistribution results of ^{68}Ga-yG5-RGD in BxPC3 and MX-1 xenograft mice at 90 min p.i.

organs / models	BxPC3	MX-1
blood	0.18±0.03	0.15±0.03
brain	0.02±0.00	0.03±0.01
heart	0.34±0.04	0.42±0.09
lung	0.85±0.06	0.86±0.21
liver	5.58±0.24	6.08±0.96
spleen	1.15±0.41	1.39±0.23
kidney	2.89±0.40	4.30±0.84
stomach	0.85±0.07	1.15±0.28
small intestine	0.68±0.34	0.76±0.18
large intestine	0.70±0.06	0.83±0.13
muscle	0.19±0.02	0.27±0.06
bone	0.56±0.08	0.87±0.19
tumor	2.54±0.44	0.42±0.11***
pancreas	0.20±0.02	0.22±0.05

(Note: each 5.55–7.4 MBq. Data are presented as mean ± SD (%ID/g, n = 4). ns P>0.05, ***P<0.001)
